# Comparability of Methods for Remotely Assessing Gait Quality

**DOI:** 10.3390/s25123733

**Published:** 2025-06-14

**Authors:** Natasha Hassija, Edward Hill, Helen Dawes, Nancy E. Mayo

**Affiliations:** 1 Center for Outcomes Research and Evaluation (CORE), Research Institute of McGill University Health Center (MUHC), Montreal, QC H4A 3S5, Canada; nancy.mayo@mcgill.ca; 2 Department of Medicine, School of Physical and Occupational Therapy, Faculty of Medicine, McGill University, QC H3A 0G4, Canada; 3 PhysioBiometrics Inc., Montreal, QC H2V 1P4, Canada; ted@physiobiometrics.com (E.H.); h.dawes@exeter.ac.uk (H.D.); 4 NIHR Exeter, Biomedical Research Centre, Medical School, University of Exeter, Exeter EX4 4QJ, UK

**Keywords:** gait analysis, Parkinson’s disease, remote assessment, wearable sensor, pose estimation, observational analysis, digital technology

## Abstract

Advancements in remote gait analysis technologies enable efficient, cost-effective, and personalized real-time assessments at home. This study aims to contribute evidence as to the comparability of gait quality metrics of three methods of remote gait assessment in individuals with Parkinson’s disease (PD): (1) observation, (2) a wearable sensor, and (3) pose estimation. A cross-sectional, multiple case series study was conducted remotely. Twenty participants submitted videos performing a modified TUG test with the Heel2Toe^TM^ wearable. Each video was analysed by six raters using the checklist specific to PD developed for this study and the MediaPipe Pose Landmarker task estimation library. The observational ratings agreed with the Heel2Toe^TM^ on detecting heel strike 64% of the time and 28.5% of the time on detecting push-off. The difference in the ranks of paired observations based on the Wilcoxon signed rank sum test between the pairs of methods compared was significant for all parameters, except for push-off when estimates from MediaPipe were compared to the ratings from the Observational Checklist, W = 86 (*p* = 0.498). A combination of digital technologies for remote gait analysis, such as wearable sensors and pose estimation, can detect subtle nuances in gait impairments that may be overlooked by the human eye.

## 1. Introduction

The restrictions placed on in-person contact, even for health-related reasons, during the COVID-19 pandemic amplified the need for remote assessments and telerehabilitation [1,2]. In physical rehabilitation, there is a strong focus on gait and mobility, traditionally assessed only in person [2,3]. Gait is the manner of walking [4]. Walking can be further classified according to capacity and performance using the ICF [5]. Capacity is what the person can do as reflected by testing parameters such as gait speed and distance walked in a fixed time in a clinical setting [6]. In measurement terminology, these tests fall under the rubric of Clinical Outcome Assessment (COA). COA can rely on different sources of information: Clinician-Reported Outcomes (ClinROs) and Performance Outcomes (PerFOs) [7,8,9]. ClinROs typically include a structured checklist [10]. PerfOs on the other hand rate a person’s performance on a standardized test such as the 10 m walking test (10MWT), Timed-Up and Go (TUG), or the 6 min walking test (6MWT). The results are interpreted as an individual’s capacity to walk or move [11]. On the other hand, performance indicates what is done in the real-world setting and is often quantified as steps per day and walking bouts [11,12]. Capacity and performance quantify walking [13]. Good gait quality is the foundation for both walking capacity and performance, making gait assessment and gait training the primary activities of rehabilitation [14,15,16].

A wide array of methods is available for assessing gait, and one method will not fit all purposes. Gait assessment is crucial for treatment planning and evaluation and uncovering mechanisms of optimal and pathological gait [17]. At one end of the spectrum are simple, inexpensive, and commonly used methods involving direct observation by an expert. At the other end are complex, costly, technologically aided methods, some of which require extensive infrastructure and specialized personnel. In a clinical setting, the direct observation method by a clinician is most widely used.

Observational methods are best suited for judging spatial gait parameters including posture, and the observer has the benefit of a three-dimensional view of the client. These parameters are rated on an ordinal scale representing the degree to which the element is optimal. Observational methods have only moderate inter-rater agreement [18] but are still widely used in a clinical setting [19].

Advances in digital recording have made monitoring gait in daily life (outside a clinic or lab) practical. The person need not walk multiple times, and the image itself can provide valuable feedback to the client. Observational methods can now be applied to gait recorded remotely.

Another rapidly advancing and evolving method for gait assessment is using technology (TechO) [7] to measure spatial, temporal, kinematic, and kinetic parameters, and these assessments can be performed using non-wearable or wearable technologies [20]. Non-wearable kinetic and kinematic technologies are considered the research gold standard [21]. Their drawbacks are that they are fixed, expensive, and need extensive infrastructure, including space and trained personnel to operate and manage data from these systems. They clearly cannot be used for remote monitoring. A newer method under the category of non-wearables for measuring many gait parameters of interest (spatial, temporal, kinematic) is based on pose estimation. This markerless motion capture technology arose from the animation industry [22] and is suitable for remote monitoring as it takes advantage of video recordings.

Wearable technologies burst into the health and fitness industries [23] owing to their availability and the miniaturization of the internal measurement units (IMUs) that detect raw inertial body angular velocity and acceleration. Wearable sensors today are lightweight, available for personal use, affordable, and can be used to collect data without disturbing daily life activities. New-generation wearables connect to smartphone devices, and IMUs are also part of every smartphone now to inform algorithms to track steps, stairs climbed, movement time, and even some aspects of gait such as stride length, unsteadiness, and asymmetry, making smartphone-based human motion assessment feasible [24]. Wearable technologies can be used clinically, for research, and for remote monitoring [25].

Of the remote methods of gait analysis, observation by an expert and pose estimation typically are possible at set intervals, and as such, they capture only a small portion of the person’s everyday gait pattern and walking performance. Wearable technologies, on the other hand, can measure gait continuously and during planned and real-world activities.

No studies have carried out a head-to-head comparison of all possible remote gait assessment methods (observation, wearable, and pose estimation), although pairwise comparisons of methods with the gold standard have been conducted. A 2021 systematic review by Follis et al. [26] reviewed 14 studies conducted on participants with lower extremity arthroplasty, comparing COA metrics using wearable sensors to traditional methods of collecting COA data typically using tests like the 6MWT or the TUG. The authors emphasized the potential of wearable sensors to provide additional insights into recovery after arthroplasty and further research on clinical changes detected by wearables. The most common head-to-head comparison was between wearable sensors and instrumented walkways [21,27]. A systematic review by Prisco et al. [27] of 32 agreement studies conducted on diverse clinical populations estimated good to moderate agreement on kinematic gait parameters but greater variability for spatial and temporal parameters. Recent studies have also compared metrics from pose estimation libraries to marker-based instrumented walkways. A study conducted by Menychtas et al. in 2023 [28], comparing 2D pose estimation libraries from OpenPose and MediaPipe Pose to the marker-based 3D motion capture system, Vicon, concluded that pose estimation libraries achieve motion tracking comparable to the fixed motion capture systems on metrics of joint angle, position of limb, and velocity of movement, although with inconsistencies in detecting smaller joint movements. Hii et al. [29] compared different pose estimation libraries in their study on marker-free gait analysis using the Pose Estimation Model in 2022 and inferred that MediaPipe Pose is the best for assessing kinematics of lower limb joints in real-world applications.

PD has been the most widely studied for these comparisons [27]. People with PD have a typical gait pattern that is well described and known to clinicians, making the gait pattern readily measurable. The gait pattern in PD is characterized by stooped posture, short shuffling steps, a centre of mass too far forward to be safe, and limited arm swing. This pattern is disabling and dangerous and negatively impacts other aspects of function and quality of life, making gait impairment the leading reason individuals with PD seek evaluation and physical rehabilitation [30].

There is a gap in knowledge about head-to-head comparisons of methods suitable for remote gait analysis and their challenges. Specifically, the purpose was to estimate, for people with PD, the extent to which values on gait metrics are comparable among (i) observational analysis by physiotherapists, (ii) wearable sensors, and (iii) pose estimation. Information from this three-way head-to-head comparison would guide clinicians and researchers to choose the best technology for their purposes.

## 2. Materials and Methods

This study is embedded in a larger ongoing implementation trial of the wearable sensor—Heel2toe^TM^ (PhysioBiometrics Inc., Montreal, QC, Canada) —as gait therapy for people with PD (ClinicalTrials.gov ID: NCT05801926). The study reported here used data from the initial assessment. Data were collected from the following materials for analysis: (i) a checklist for observers to rate elements of gait that are specific to PD; (ii) remotely recorded videos of adequate quality for observing gait; (iii) a wearable sensor that detects the angular velocity of the ankle during the gait cycle—Heel2Toe^TM^ wearable; (iv) a pose estimation library—MediaPipe Pose.

### 2.1. Sample

All participants gave their written informed consent in agreement with the Declaration of Helsinki, and the study protocol was approved by the Institutional Review Board of the Faculty of Medicine and Health Sciences, McGill University, on 31 August 2022 (A05-B37-22A).

The trial participants included people with self-reported mild to moderate gait deficits equivalent to Hoen and Yahr grade ≤ 3 and who were able to perform a modified TUG test (walking a minimum of 15 steps instead of the standard 3 m) without losing balance or freezing without recovery as observed on a personally recorded video. Participants received a comprehensive list of instructions for recording the video, with an example video recorded by the research team as a reference. Participants were asked to record both lateral and frontal views performing the modified TUG test wearing the Heel2Toe^TM^ wearable sensor between 30 min and 2 h after taking their medication (Levodopa/Carbidopa).

### 2.2. Measures

(a) Observational Checklist

An observational checklist (see Appendix A) was adapted for use in this study based on existing checklists described in the literature [31,32,33,34,35,36]. The checklist comprised 34 different gait parameters (items) rated by six raters to outline the degree to which each was optimal. Each item in the checklist was rated on a binary or three-point ordinal scale that, when summed, yielded a total score of 35 points, where a higher score indicated better gait quality. The checklist was iteratively tested and revised based on the experience of nine different gait experts. Videos of adequate quality were analysed by six randomly assigned raters from a diverse group of ten experienced physiotherapists. Raters showed an average agreement of 86.4% across all items. There was 100% agreement on 3 out of 34 items. For each item with less than perfect agreement, a consensus rating was chosen after review by two expert raters. The absolute intraclass correlation (ICC) across raters was 0.78.

(b) Heel2Toe^TM^—wearable sensor

**Figure 1:**
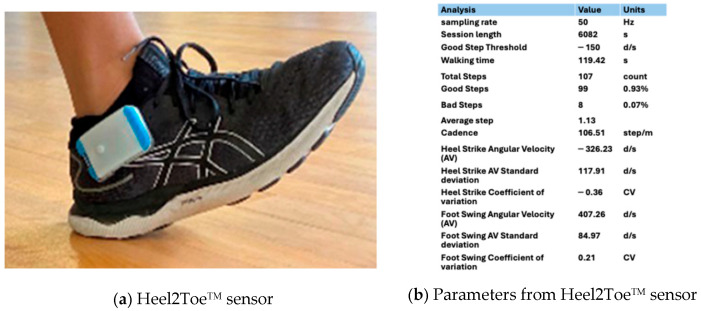
(**a**) The Heel2Toe^TM^ sensor on the right foot; (**b**) the parameters displayed on the application.

The Heel2Toe^TM^ wearable is a commercial product of PhysioBiometrics Inc. It is a small, wireless, inexpensive, and lightweight wearable sensor that clips to the side of the shoe, as shown in Figure 1a. The gyroscope in the sensor measures in real time the angular velocity of the ankle in the sagittal plane during each gait cycle. The angular velocities are negative with clockwise rotation and positive with counterclockwise rotation. An algorithm generates metrics related to heel strike and toe push-off, which are clockwise rotations, and foot swing, which is a counterclockwise rotation. The salient parameters are shown in Figure 1b and fully described in Appendix B. The version of the Heel2Toe^TM^ sensor used in this study transmitted raw data from the sensor to an application on an Android phone via Bluetooth and saved them on a cloud server.

Heel2Toe^TM^ can be used for both assessment and training purposes as it provides feedback for an optimal step. The development of the hardware and algorithm of the Heel2Toe^TM^ has been reported [37,38]. The algorithm has a step classification accuracy of 92.7%, with a sensitivity and specificity of 84.4% and 97.5%, respectively. The version of the Heel2Toe^TM^ wearable used for this group of participants used a Bluetooth connection from the sensor to an app on the smartphone. The data are transmitted to the google cloud server and analysed using a proprietary algorithm to detect steps and quantify angular velocities. The Heel2Toe^TM^ has been successfully tested among individuals with PD, demonstrating feasibility for home use and strong efficacy potential [39,40].

(c) Analysis of video-recorded gait using MediaPipe Pose

**Figure 2:**
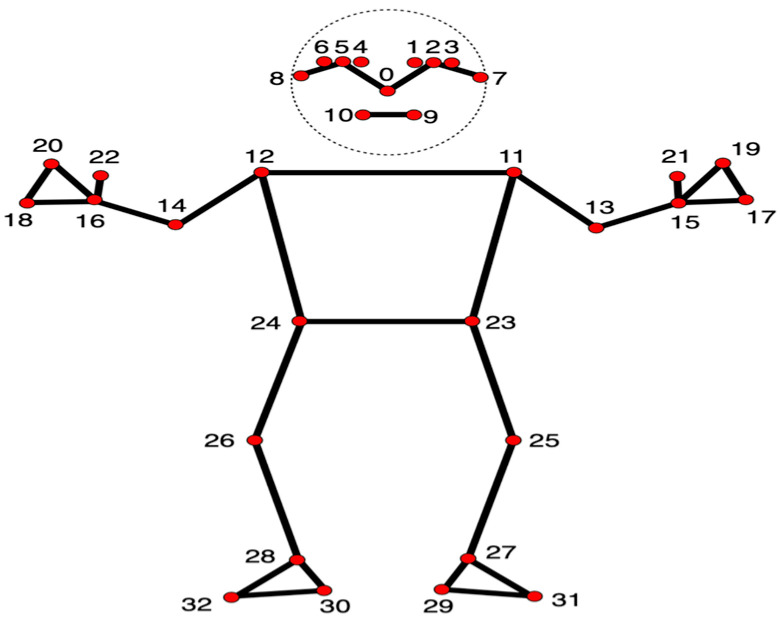
Anatomical landmarks detected using MediaPipe Pose (this image has been reproduced from Google AI for Developers, Mountain View, CA, USA).

The MediaPipe Pose Landmarker task, developed by Google, was used to analyse 20 gait videos using the Python 3.10.0 PyPI package. The Pose Landmarker model uses a convolutional neural network to map human pose by estimating 33 three-dimensional (x, y, z) landmarks, also called anatomical landmarks, in real time, as shown in Figure 2. Compared to other open-source libraries, MediaPipe shows high accuracy in mapping anatomical landmarks of the lower limb [29]. Furthermore, MediaPipe Pose has been tested for reliability in gait analysis and the detection of motor impairments in PD [41,42]. Studies conducted so far using the MediaPipe Pose library for gait analysis have reported detecting heel strike, push-off, step length, stance time, swing time, and double support time [29,43]. As pose estimation is less explored, automated models for detecting only a few gait parameters have been developed, and clinically relevant normative values are not available.

**Figure 3:**
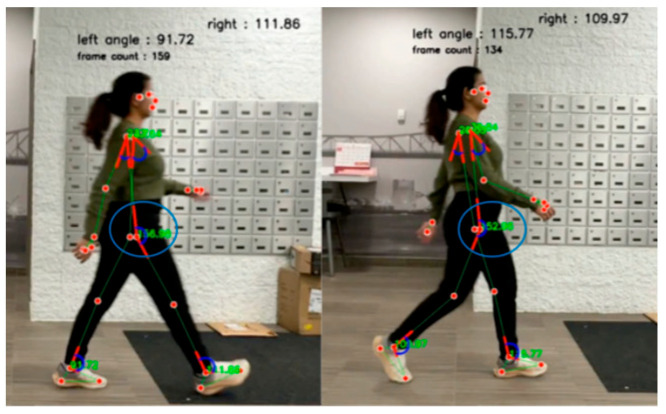
Initial testing for extracting gait parameter estimates using MediaPipe Pose from a video of a person with optimal gait.

To meet the need for detecting a broader range of temporal, spatial, and kinematic gait parameters for a comprehensive analysis best suited for clinical interpretation, a program inspired by the studies on pose estimation [42,44,45] was customized specifically to the observational checklist developed in this study. We aimed to identify as many gait parameters as possible. In the process, each gait parameter was first defined, and the relevant anatomical landmarks were listed. After running multiple experiments attempting to estimate angles and distances on the videos shared by the participants, we were limited to estimating 5 out of the 31 parameters from the checklist. This limitation was due to challenges of a lack of an estimate of the ground, inconsistencies in the distance covered during the modified TUG test, fluctuating angles of the camera, inconsistent resolution, and unsteady recording, leading to variability in the video frames captured. All videos were analysed in the MP4 format at 60 frames per second. This tailored program was first tested on a video with no gait impairments, as shown in Figure 3. The parameters we were able to estimate are (i) heel strike and push-off in degrees/second (°/s)—values corresponded to the most optimally viewed frame of heel strike and push-off on the video; (ii) swing at the hip in degrees (°)—values corresponded to the mean ± SD representing a change in angles at the hip during one gait cycle identified by frames on the video; and (iii) forward and backward arm swing in degrees (°)—values corresponded to the most optimally viewed frame of forward and backward arm swing on the video. For this analysis, it was not possible to automate the model to identify gait parameters, and thus a gait expert (NH) chose the best optimally viewed frame for analysis. The libraries and modules used in the program are fully described in Appendix C.

Table 1 presents gait parameters acquired from the three methods and illustrates the commonality of parameters across methods.

### 2.3. Analysis

Two types of analyses were carried out. The first was on the agreement between the observational checklist and values from the Heel2Toe^TM^ wearable. To match the qualitative categories from the observational checklist, values obtained from Heel2Toe^TM^ for heel strike and push-off were categorized as excellent, very good, good, fair, or poor; values for foot clearance and cadence were dichotomized. Categories were based on typical values generated from a sample of 88 students in health professional programs at McGill (see Table 2). The crude agreement was calculated for each of the four parameters. For the four metrics and a sample size of *n*, the number of persons measures a total of 4 × *n*. The agreement rate per person-measure and the associated 95% confidence interval (CI) were calculated using RStudio^®^ (2024.09.0) statistical software. The second analysis related estimates of angle parameters (angular velocity or joint angle) from MediaPipe Pose to the categorical ratings from the observational checklist and to the angular velocity from the Heel2Toe^TM^ wearable. As the parameters compared across all three methods were on different measurement scales, the participants were ranked for each parameter, from highest to lowest, on the respective measurement scales. The differences in these rankings were compared using the Wilcoxon signed rank sum test. A significant *p*-value indicates a substantial difference between the median values of each pair of methods being compared. Finally, measured angular velocities from MediaPipe Pose and the Heel2Toe^TM^ wearable for heel strike and push-off were grouped based on ratings from the observational checklist and compared using *t*-tests. For these comparisons, the *t*-test was used as an estimator of effect size rather than a difference between means [46].

### 2.4. Sample Size

The sample size was estimated during the planning stages based on having a feasible sample ranging from 14 to 20 participants and calculating a person-measure agreement rate based on a minimum number of participants of 14 and 4 overlapping measures among the different methods, yielding 56 person-measures. With a hypothesized agreement rate of 0.5, the associated 95% CI ranged from 0.33 to 0.72.

## 3. Results

Twenty-seven people submitted videos, and none were excluded based on safety considerations. Seven people submitted videos of insufficient quality (whole person could not be observed, incomplete task performance, blurry or shaky) for any of the gait quality assessments. The remaining 20 participants had videos suitable for observational gait analysis and MediaPipe Pose. Of the 20 participants, 14 met the technology readiness criteria for home use of the Heel2Toe^TM^ wearable.

The characteristics of the participants in terms of demographics and quality of video submitted at the initial assessment when recruited for the implementation trial are shown in Table 3.

Table 4 presents the agreement on the qualitative categories between the observational checklist and the Heel2Toe^TM^ wearable: heel strike, push-off, foot clearance, and cadence. The optimal values of these kinematic parameters are given in Table 2. The first cell indicates that there was agreement at the optimal/excellent level on heel strike for 3 of the 14 comparisons, and the overall crude agreement was 9/14 (64%) (CI: 0.39, 0.89). For push-off, the crude agreement was 28% (CI: 0.04, 0.52); for foot clearance, the crude agreement was 35.7% (CI: 0.12, 0.61); for cadence, the crude agreement was 85% (CI: 1.01, 2.71). Finally, the overall agreement across all four parameters was 53.5% (CI: 0.41, 0.66).

Table 5 presents values on matching parameters estimated from MediaPipe Pose and rated using the observational checklist and also from the values recorded using the Heel2Toe^TM^ sensor. Given that common parameters from all three methods were measured on different metric system units, the Wilcoxon signed rank test was conducted to see if paired common parameters were ranked the same. The difference in the ranks of paired observations between the pairs of methods compared was significant for all parameters except for push-off when estimates from MediaPipe were compared to the ratings from the observational checklist, W = 86 (*p* = 0.498).

Table 6 presents the differences between optimal and weak parameters as rated by expert viewers using the observational checklist when the parameters were assessed using each of the TechOs. For heel strike, there were 6 people rated optimal by viewers and 14 rated weak. The *t* value for the difference between optimal (−222.8°/s) and weak (−156.5°/s) was low (1.6). This was also true for values of push-off. The corresponding *t* values when measured using Heel2Toe for these two groups were also low. For people rated with optimal heel strike observationally, the *t* value for heel strike between MediaPipe and Heel2Toe sensor was low. However, there were strong effect sizes when heel strike was judged weak (*t* = 2.6) and when push-off was judged both optimal and weak (*t* = 6 and 3.3, respectively).

## 4. Discussion

This study was motivated by the need for remote assessment, and hence, the methods compared are not commonly integrated into the clinical setting. Remote assessments can be conducted by analysing videos recorded using a single camera through expert observation, pose estimation, or real-time analysis using wearable sensors [48].

A scoping review of the status of clinical gait assessment published in 2022 by Hulleck et al. [49] highlighted a gap between the technology available and current clinical practices, given most clinicians rely on methods of observation or performance for gait assessment due to the lack of clinical interpretability of data obtained from TechOs. To bridge this gap and provide comparable data, an observational checklist specific to gait assessment in PD was developed for this study and tested as one of the methods for evaluating gait from videos. This approach also allowed for the application of pose estimation to the same videos. Additionally, a wearable sensor was used for real-time analysis. To our knowledge, this is the first time that these three methods have been directly compared using data and videos from individuals with PD in their home environment, demonstrating how telerehabilitation might work for remote assessment.

A systematic review by Ridao-Fernández et al. in 2019 [34] reported the reliability of 18 observational measures assessing spatial and temporal gait parameters as poor to fair. Furthermore, a study by Krebs et al. in 1985 [33] reported an ICC of 0.73 for the Observational Kinematic Analysis measure. This is similar to the ICC calculated for the observational checklist in our study, which was calculated as 0.78, capturing both spatial–temporal and kinematic parameters, suggesting it to be sufficient for treatment planning but not adequate for estimating individual change [50]. This could be due to possible challenges related to a lack of sufficient optimization of the measure, insufficient training of the raters, ambiguity in phrasing questions positively/negatively, the time-consuming nature of the checklist, inconsistencies in rating one side instead of both, and poorly recorded videos.

Our results show that observational gait quality ratings differed from those obtained using MediaPipe except for push-off and were also different from those obtained using the Heel2Toe wearable sensor (see Table 5). We also showed that observational ratings of quality (optimal and weak) did not correspond to the values obtained from the TechOs (see Table 6). There were also differences across TechOs as values from MediaPipe are estimates of angular velocities from angular changes over time, while values from Heel2Toe are measured directly from IMUs embedded in the device.

A systematic review by Follis et al. in 2020 [26] compared traditional observational methods, including Performance Outcomes (PerfOs) and Clinician-Reported Outcomes (ClinROs), to wearable sensors for assessing functional change after lower limb arthroplasty. The review reported varying levels of agreement between wearables and observational methods, ranging from poor to moderate correlations. It highlighted the holistic nature of observational methods for assessing gait, in contrast to the limited parameters obtained from a single sensor, despite the sensor’s ability to identify subtle biomechanical nuances overlooked by traditional methods. This challenge can be addressed by using multiple sensors to evaluate different aspects of gait. In our study, the highest degree of agreement between the observational checklist and the data from the Heel2Toe™ wearable was on cadence (92%), which can be used to detect variability in walking speed. Moderately high agreement was found for heel strike (62%), while poor agreement was observed for other gait parameters: push-off (28.6%) and foot clearance (35.7%) (see Table 4). One major challenge while recording data using the Heel2Toe™ was the occasional loss of connectivity signal due to an unstable Bluetooth connection and the wearable’s selective connection with Android operating systems. These issues occasionally required participants to re-record sessions, and any disconnection during a session resulted in the data not being saved to the cloud. These issues have been addressed in newer versions of the Heel2Toe™.

A study by Ramesh et al. in 2023 [44] comparing an observational method of gait assessment with pose estimation reported a strong correlation of 0.8 between the Edinburgh Visual Gait Score, which captures spatial and temporal parameters, and OpenPose, a pose estimation library. This contrasts with our findings, where the only parameter showing agreement between the observational checklist and MediaPipe was push-off; there was no agreement on other overlapping measurements such as heel strike, hip swing, and arm swing (see Table 5). One of the greatest challenges is the absence of scores verified by ground truth. Additional challenges included the lack of robustness of the model to variations in lighting, camera positioning and angles, resolution, and other objects in the video frame. The cumbersome nature of frame-by-frame analysis due to the lack of automation of the model and the need for different views (frontal for temporal parameters and lateral for spatial parameters) complicates the recording of gait videos. Due to the small size of the foot, variability in camera angles and inconsistencies in video frames led to more variability in identifying gait metrics at the ankle in comparison to the hip and the shoulder as they are larger and have better visibility on camera. In addition, contrary to our hypothesis, the minimum and maximum angles at the ankle from the individual manually identified gait cycles did not correspond to a heel strike or push-off. For example, the maximum angle recorded was at the foot flat phase of the gait cycle instead of the push-off phase. Additionally, the relative distances varied with the angle and distance of the camera from the person in the video, which could have been avoided if the camera was mounted and the person walked withing the range of field visibility of the camera lens. As the videos were recorded in different settings (at home, in shopping malls, on the street), there was lack of ground estimate, which further limited extraction of gait parameters using the existing pose estimation model. These factors would have had an even greater influence on measurement error with a fully automated model when used in a real-world scenario. Recording both views may not always be feasible in all scenarios [51]. Furthermore, the lack of open-source automated libraries for pose estimation makes it challenging to integrate these technologies into clinical settings, posing computational difficulties for clinicians in their current format. Nonetheless, these technologies can detect subtle nuances missed by the human eye, offering promising possibilities for early detection of impairment during an assessment from the comfort of one’s home [47].

While comparing pose estimation to wearable sensors, a study by Masataka Yamamoto et al. in 2022 [52] reported a strong correlation of 0.89 in detecting mean joint angles between the two methods in the lateral view. Our study found no agreement when the difference in ranks from angular velocity values obtained from MediaPipe and Heel2Toe^TM^ were compared, as shown in Table 5, possibly due to the challenge of varying camera angles and clutter in the environment, which affected the tracking of key landmarks. These are challenges that would commonly exist in a scenario of a remote clinical setting. Nonetheless, both TechOs were able to differentiate between ratings from the observational checklist. When grouped based on those ratings, matching pairs showed an agreement (see Table 6).

A limitation of this study is the small number of subjects, only 20, and not everyone could be assessed on all three methods. However, given the low amount of agreement, a better use of resources would be to optimize the methods for remote assessment before assessing more people. A single camera 2D setup using a smartphone was used to record videos in this study due to its easy access at home. It is also a setup commonly used in clinical environments and for remote monitoring of gait impairments, although its scope for gait assessment is limited [53]. However, reconstruction of two-dimensional (2D) videos into three-dimensional (3D) views to capture subtle rotational components of gait missed in the 2D view could improve its scope and precision for remote assessments. However, studies have shown moderate to strong correlation between 2D and 3D views in the frontal and sagittal planes [54,55]. As pose estimation becomes more robust for human motion, it could emerge as a very useful tool when used in combination with wearable sensors for remote assessment.

A perceived limitation could be that three of the co-authors N.E.M., E.H., and H.D., are officers of PhysioBiometrics Inc., the company that commercializes the Heel2Toe wearable. N.E.M., the principal investigator on the research project, was not involved with the analysis but guided N.H. on interpretation. E.H. guided N.H. on MediaPipe Pose but did not link the results to those from the Heel2Toe sensor. H.D. reviewed the text of the manuscript only. Thus, the roles of the in-conflict co-authors had no effect on the results. The conflict was declared in the consent form, making it clear that the principal investigator, N.E.M., potentially would benefit from the results of this study.

## 5. Conclusions

The results of this study support that the wearable sensor has the most promise for regular clinical use and at-home use as patients can use it themselves, and the data are readily interpretable by both the patient and clinician. The newest version of the Heel2Toe™ records data directly onto an SD card and can be transmitted for visualization and storage on to a computer, tablet, or smartphone via a standard USB cable. It does, however, provide a limited set of gait parameters. The observational checklist is too time-consuming and unreliable for individual assessment of changes. MediaPipe pose requires too much expertise for the average clinician to use regularly but certainly provides rich data when used optimally. Advances in automating the program for estimating a variety of gait parameters and in image processing could change its real-world applications.

## Figures and Tables

**Table 1:** The commonality of gait parameters across the three different remote gait assessment measures.

Gait Parameter	Observational Checklist	Heel2Toe^TM^ Wearable	MediaPipe Pose
Freezing	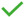	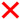	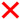
Base of support	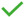	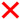	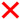
Poor foot clearance	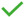	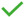	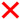
Unsteady while walking	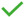	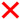	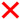
Variable pace dynamics	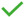	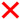	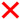
Heel strike	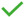	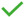	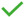
Push-off	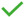	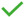	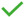
Cadence	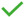	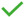	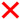
Swing at the hip	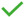	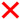	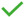
Gait symmetry	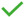	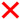	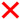
Symmetry of arms while swinging	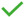	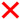	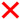
Forward and backward arm swing	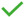	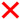	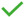
Posture	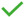	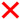	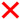
Tremor	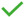	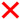	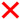
Dyskinesia	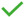	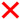	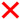
Rotated trunk	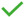	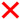	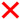
Ability to pivot	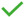	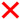	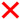

**Table 2:** Categories for gait parameter estimates that were recorded using Heel2Toe^TM^ wearable based on 88 healthy students.

Parameter/ Category Ratings	Excellent	Very Good	Good	Fair	Poor
	|Maximum|	25th or 75th Percentile	Median	25th or 75th Percentile	|Minimum|
Heel strike (°/s)	−400 to <−320	−320 to <−280	−280 to <−200	−200 to <−120	<−120
CV%	10 to <20	20 to <25	25 to <30	30 to <50	≥50
Push-off (°/s)	−600 to −481	−480 to −421	−420 to −301	−300 to −121	−120 to 0
CV%	5 to <15	15 to <25	25 to <30	30 to <50	≥50
Foot clearance (°/s)	600	400	360	340	200
**CV%**	5 to <10	10 to <15	15 to <20	20 to <30	≥30

For heel strike and push-off, a negative number indicates a stronger step; conversely, for foot clearance, a positive number signifies a stronger step.

**Table 3:** Characteristics of 20 participants according to method of data acquisition.

Variables	Observational Checklist/ Media Pipe Pose (*n* = 20)	Heel2Toe^TM^ Wearable (*n* = 14)
Age: Median years (range)	69 (56–80)	69 (57–75)
Sex: Men	10 (50%)	7 (50%)
Falls in the past 12 months *n* (%)		
0	9 (45%)	5 (36%)
1–2	8 (40%)	6 (43%)
3–5	2 (10%)	2 (14%)
6+	1 (5%)	1 (7%)
Cognition		
Symbol Digit Modalities Test (SDMT) norm ~50 Median (Range) *	32 (18–53)	34 (18–53)
HRQL		
EQ-5D Descriptive System		
Problems walking about	16 (80%)	12 (86%)
Problems washing/dressing	5 (25%)	3 (21%)
Problems doing usual activities	16 (80%)	10 (71%)
Pain/discomfort	18 (90%)	12 (86%)
Anxiety/depression	11 (55%)	7 (50%)
Self-rated health: median/100 (range)	73 (19–90)	78 (50–90)
Preference-Based Parkinson’s Index		
Descriptive System		
Trouble falling back to sleep	7 (35%)	6 (43%)
Difficulty remembering	4 (20%)	4 (29%)
Walking aid/assistance	2 (10%)	1 (7%)
Fatigue needing rest during the day	3 (15%)	1 (7%)
Happy/positive only sometimes or rarely	2 (10%)	1 (7%)
Shaking/ tremor interfering with their activities	7 (35%)	4 (29%)
Any difficulty using hands for activities of daily living	13 (65%)	10 (71%)
Video Quality		
Excellent	1 (5%)	0
Good	7 (35%)	7 (50%)
Fair	8 (40%)	5 (36%)
Poor	4 (20%)	2 (14%)

* Average SDMT scores for healthy adults aged 40–59 years is 45–50; for those 60 years and above, it is 40–50 [47].

**Table 4:** Agreement on the quality of common gait parameters between the observational checklist and the Heel2Toe^TM^ wearable.

Observational Checklist		Heel2Toe^TM^ Wearable			
Heel Strike	Excellent/Very Good	Good	Fair/Poor	Total	Crude Agreement (95% CI)
2 (Optimal)	3	2	0	5	64.3% (38.8%, 83.7%)
1 (Weak)	2	6	1	9	
0 (Poor)	0	0	0	0	
Total	5	8	1	14	
Push Off	Excellent/Very Good	Good	Fair/Poor	Total	Crude Agreement (95% CI)
2 (Optimal)	2	2	0	4	28.6% (11.7%, 54.7%)
1 (Weak)	3	2	4	9	
0 (Poor)	0	1	0	1	
Total	5	5	4	14	
Foot Clearance	Excellent/Very Good	Good/ Fair/Poor	Total	Crude Agreement (95% CI)	
1 (Not Poor)	3	1	4	35.7% (16.3%, 61.2%)	
0 (Yes, Poor)	8	2	10		
Total	11	3	14		
Fast Cadence	Slow/Purposeful/Moderate/Brisk	Fast	Total	Crude Agreement (95% CI)	
1 (Absent)	12	1	13	92.9% (68.5%, 98.7%)	
0 (Present)	1	0	1		
Total	13	1	14		
Overall	20	10	56	53.6% (40.7%, 66.0%)	

Agreement pairs are highlighted in green.

**Table 5:** Wilcoxon signed rank sum test for pairwise comparisons of parameters estimated from MediaPipe Pose and matching parameters rated using the observational checklist and the values recorded using the Heel2Toe^TM^ wearable sensor.

Gait Parameter	MediaPipe—Observational Checklist (*n* = 20)		MediaPipe—Heel2Toe^TM^ Wearable (*n* = 14)	
	W	*p*-Value	W	*p*-Value
Heel Strike	7	0.0002	93	0.0085
Push-Off	86	0.498	102	0.0006
Swing at Hip	206	0.0001	-	-
Forward Arm Swing	210	0.00008	-	-
Backward Arm Swing	153	0.0003	-	-

**Table 6:** A summary of the results from the *t*-tests for angular velocity values from MediaPipe Pose and the Heel2Toe^TM^ wearable grouped by binary ratings for heel strike and push-off obtained from the observational checklist.

Grouped by Observational Ratings	MediaPipe		Heel2Toe^TM^ Wearable			
	*n*	Mean (SD)	*n*	Mean (SD)	*n*	*t* Value **
Heel Strike						
Optimal	6	−222.8 (53.8)	5	−290.6 (75.1)	5	1.5
Weak	14	−156.5 (134.0)	9	−241.8 (65.0)	9	2.6
*t* value *		1.6		1.2		
Push-Off						
Optimal	5	51.0 (276.3)	4	−437.0 (119.1)	4	6
Weak	15	−79.0 (192.0)	10	−361.6 (110.8)	10	3.3
*t* value *		0.9		1.1		

* *t*-test of unequal variance; ** paired *t*-test.

**Figure A1:**
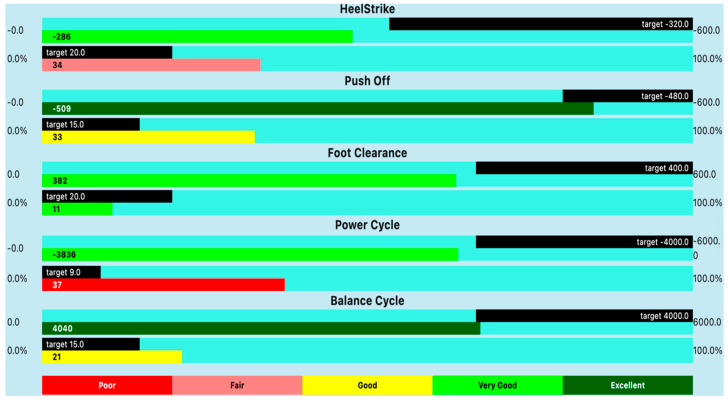
An example of a dashboard generated using parameters recorded from the Heel2Toe^TM^ wearable.

**Figure A2:**
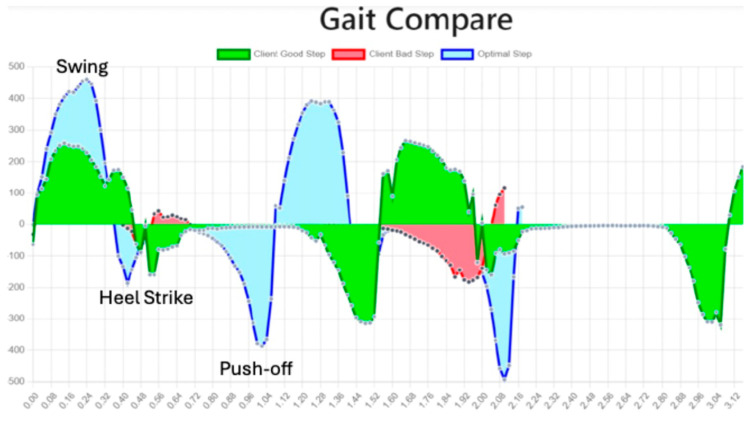
Gait cycle from the z-axis of the gyroscope.

**Figure A3:**
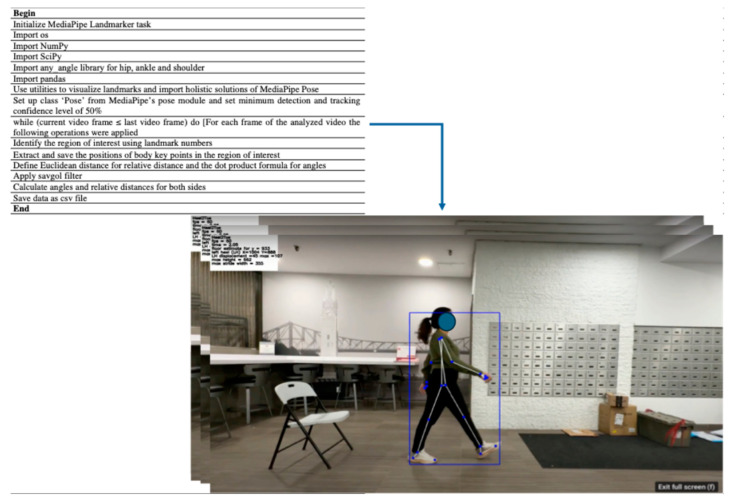
Outline of program and initial output.

**Figure A4:**
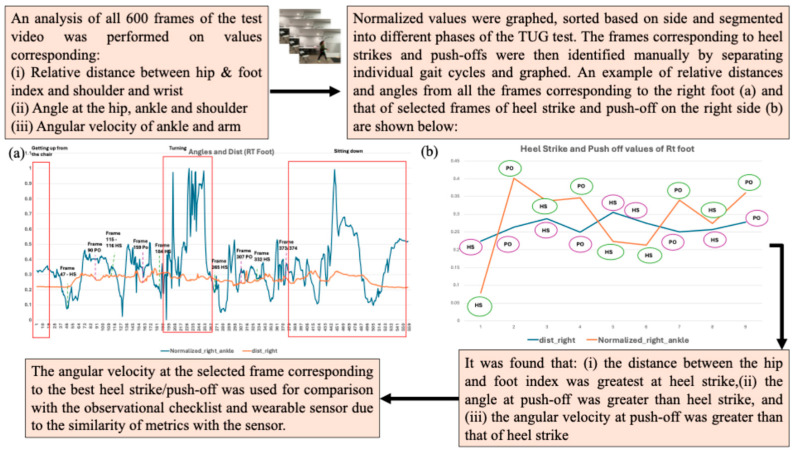
Overview of workflow for estimating heel strike and push-off.

**Figure A5:**
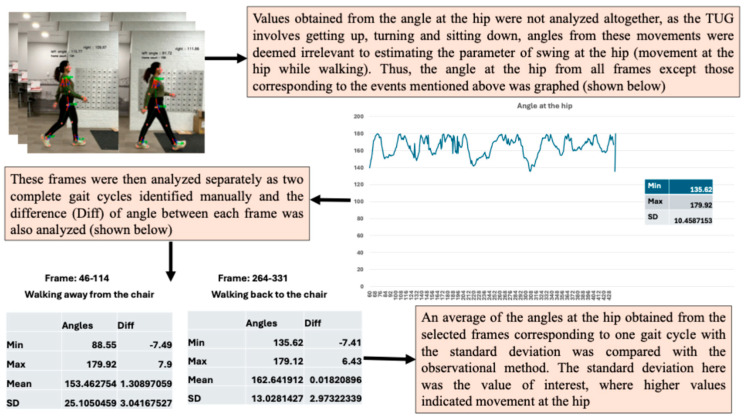
Overview of workflow for estimating swing at hip.

## Data Availability

Data supporting the reported results can be found at https://escholarship.mcgill.ca/concern/theses/mw22vc13j (accessed on 8 January 2025).

## Appendix A

**Table A1:** Final checklist used for rating 20 videos by raters.

Gait Parameters	Description	Classifier
**Getting up from the chair**		
Freezing while getting up	We are looking to see if the participant experiences the sudden inability to move despite the intention to while getting up from the chair.	0 Present 1 Absent
Needs arms	Does the participant use the support of the armrest/side of the chair or do they place their arm on their thighs or knees as support to get up from the chair.	0 Yes 1 No
More than 1 attempt to get up from a chair	Does the participant try getting up more than once from the chair due to unsuccessful attempt/attempts.	0 Yes 1 No
**Walking**		
Narrow base of support	Base of support (BOS) is the area formed by contact points of the feet with the ground. For example when you stand with your feet shoulder-width apart, your BOS is the area covered between the feet. BOS changes with movement. A barrow BOS while walking can be noticed if there is crossing of the feet. A normal BOS is slightly less than shoulder width, and the feet would be in line with the width of the hips.	0 Yes 1 No
Freezes while walking	Freezing is the sudden inability to move despite the intention to. We are looking to see if the participant experiences this sudden inability to move while walking.	0 Present 1 Absent
Looks at feet	Does the participant look down at their feet while walking instead of looking forward.	0 Yes 1 No
Scuffs foot/Poor foot clearance	Does the participant drag their foot (not lift it sufficiently to place it on the ground)? Please note you could select ‘Yes’ even if there is poor foot clearance on one side.	0 Yes 1 No
Unsteadiness while walking	Experiences short instances of losing balance while walking without falling.	0 Yes 1 No
Pace dynamics while walking		
Variable pace while walking	A gait pattern where the pace of walking may fluctuate, with periods of slower movements interspersed with rapid uncontrollable acceleration. Does the participant experience bradykinesia and festination?	0 Yes 1 No
**Gait Parameters**		
Heel strike	A heel strike is also known as initial contact. It is a phase of the gait cycle that occurs when the heel touches the ground while walking. Hint: When viewed anteriorly, a complete visual of the sole when the heel contacts the ground indicates an optimal heel strike.	2 Optimal 1 Weak 0 Absent
Push-off	The push-off phase involves the propulsion of the body forward as the foot pushes off the ground to initiate the swing phase of walking. Hint: A complete visual of the sole during the phase indicates an optimal push-off when viewed posteriorly.	2 Optimal 1 Weak 0 Absent
Fast cadence	Does the individual have an abnormal increase in speed or frequency of steps leading to a shuffling gait?	0 Present 1 Absent
Swing at hip	The swing phase is when the leg is not in contact with the ground and actively moves forward to prepare for the next step. It is characterized by a series of movements at the hip joint including hip flexion, extension, and abduction/adduction which facilitate foot clearance and forward progression.	1 Optimal (Step is initiated with an almost straight knee) 0 Weak (Excessive movement of the knee from flexion to extension)
**Symmetry**		
Gait symmetry	Gait symmetry refers to the equality between the movements of the left and right limbs during walking. This will be shown through differences in step length, time of foot contact with the ground, and amplitude of joint movement.	1 Present 0 Absent
Symmetry of arms while swinging	The symmetry of the arms while walking refers to the equality between the arm swings on the right and left sides.	1 Present 0 Absent
Coordination of the arms with the legs	This refers to balanced and alternating movement of the arms in coordination with the movement of the legs, contributing to a smooth and efficient gait pattern.	1 Present 0 Absent
**Arm Swing**		
Forward arm swing	The forward arm swing involves rotational movement of the arms alongside the body where the arm swings forward, crossing the midaxillary line. Predominantly, forward arm swing is greater than backward arm swing.	2 Optimal 1 Reduced 0 Absent
Backward arm swing	The backward arm swing entails rotational movement of the arms alongside the body where the arm swings backward, crossing the midaxillary line.	2 Optimal 1 Reduced 0 Absent
**Posture**		
Flexed at hip	Forward leaning of the trunk posture predominantly seen in those with PD is associated with flexion at the hip. This means the hips are bent or flexed forward, reducing range of motion at the hip and contributing to an overall stooped appearance.	0 Yes 1 No
Rounded shoulders	Rounded shoulders or slouched posture is a common postural issue where the shoulders are positioned forward, causing the upper back to appear rounded.	0 Yes 1 No
One shoulder lower than the other	The shoulders may not be at the same level due to differences in muscle tone on either side of the body, which could lead to the asymmetric presentation of the shoulders.	0 Yes 1 No
Forward lean of the head	It is a common postural misalignment, often evident when the head is positioned forward compared to the shoulders and the ear aligns ahead of the shoulder rather than directly over it.	0 Yes 1 No
**Tremor**		
Tremor	Arm tremors typically occur at rest and usually involve rhythmic shaking or oscillatory movements of the forearms/wrists/hands.	0 Present 1 Absent
Dyskinesia	Dyskinesia is characterized by involuntary and uncontrolled movements that are often exaggerated or excessive. These movements can be jerky, writhing, or twisting, typically affecting the limbs, face, or trunk. Dyskinesia can manifest as chorea (rapid, jerky movements), dystonia (sustained muscle contractions causing twisting or repetitive movements), or athetosis (slow, writing movements).	0 Present 1 Absent
**Trunk while walking**		
Rotated	The trunk would be twisted or rotated towards the affected side while walking due to differences in tone and muscle weakness.	0 Yes 1 No
Anteroposterior movement of the trunk	Anteroposterior movement of the trunk refers to the normal forward and backward motion of the upper body during walking. For example, when we walk, our trunk naturally sways back and forth in coordination with the movement of our legs.	0 Present 1 Absent
**Turning**		
Unable to pivot	Instead of pivoting on one foot (active rotation of the foot around its own vertical axis) to execute the turn, the individual may take small steps in a circle.	0 Yes 1 No
**Sitting on the chair**		
Unable to turn and sit in one motion	Unable to turn and sit in one motion. Takes multiple small steps (more than three steps while turning to sit).	0 Yes 1 No
Freezes while trying to sit on the chair	Sudden inability to move despite the intention to. We are looking to see if the participant experiences this sudden inability to move while trying to sit on the chair.	0 Yes 1 No
Uses arms as support to sit	Does the participant use the support of the armrest/side of the chair or place their arm on their thighs or knees to sit on the chair?	0 Yes 1 No
Unable to control the descent	The participant uses the support of both arms or one arm to control the descent on the chair or drops the entire body weight instantly.	0 Yes 1 No

## Appendix B

Parameters extracted from the Heel2Toe^TM^ wearable have been explained below.

**Table A2:** Gait parameters recorded using the Heel2Toe^TM^ wearable.

Gait Parameter	Description
1 Angular velocity at heel strike (HeelStrikeAV)	The speed at which the foot moves from dorsiflexion when the heel strikes the ground to neutral when the foot is flat on the floor. It is measured in °/s. It is the clockwise movement of the ankle at the pivot which is recorded as a negative value by the sensor.
2 Angular velocity at push-off (HeelOffPowerAV)	The speed at which the heel lifts off the floor to propel the body forward. It is a clockwise movement around the pivot point of the ankle and is recorded as a negative value by the sensor.
3 Power cycle (PowerPhaseAUCAV)	The phase of the gait cycle from heel strike to push-off that essentially generates the power to propel the body forward. It is calculated by summing the areas under the zero line on the graph. It is recorded as a negative value and is measured in (°/s)^2.^
4 Angular velocity of foot clearance (FootSwingAV)	The speed at which the foot pivots around the ankle joint from plantarflexion at push-off to dorsiflexion when the leg is preparing to position the foot to make a heel strike. A certain angular speed is needed to clear the toes from the ground, or the person can stumble and fall. As the movement is counterclockwise, the value is positive.
5 Balance cycle (BalancePhaseAACAV)	The swing phase of the gait cycle when one foot is in the air swinging forward and the other foot is on the ground. The height and duration of the swing create an area measured in (°/s)^2^. The magnitude of this area depends on the person being able to stand on one leg, termed single leg stance.
6 Coefficient of variation HeelStrikeAVCV HeelOffPowerAVCV FootSwingAVCV PowerPhaseAUCAVCV BalancePhaseAACAVCV	The sensor generates gait metrics for each step. When the person takes many steps, as in a walking test, the average value is one summary metric as well as the variability (standard deviation) around the mean. The coefficient of variation is the ratio of the standard deviation of angular velocity to the average value, indicating how consistently a person walks.

## Appendix C

OpenCV library, SciPy library, NumPy, Pandas, and the ‘os’ module. The parameters were estimated using the method of (i) the relative distance, (ii) the angle at the joint and angular velocity, or (iii) a combination of relative distance and angles. Relative distances were measured in pixels, with angles in degrees and angular velocities. The parameters of heel strike and push-off (landmarks for relative distance used were 24,32 and 23,31 while those for the angle at the ankle were 26, 28, 32 and 25, 27, 31) were estimated using the angular velocity method in degrees/second (°/s). Arm swing was estimated using a similar method (landmarks used to detect relative distance were 12,16 and 11,15 and those used to measure the angle at the shoulder were 14, 12, 24 and 23, 11, 13). Swing at the hip was calculated according to the angle in degrees (°) at the hip (using landmarks 26, 24, 12 and 25, 23, 11). The angle at the joint was estimated using the dot product formula for two vectors,

*a .b* = ‖*a*‖*a .b* = ‖*a*‖·‖*b*‖cos θ,

where θ is the angle between *a* and *b*, and ‖a‖·‖b‖ is the magnitude of the vectors a and b. The angular velocity was estimated using the formula

*ω* = Δ*θ*/Δ*t* = Δ*θ* × *fps*,

where *ω* is the angular velocity, Δ*θ* is the change in angle calculated using Δ*θ* = *θ*n + 1 − *θ*n, Δ*t* is the time interval between frames, and Δ*t* = 1/*f**p**s*, where fps is frames per second. Relative distances were estimated using the Euclidean distance formula, where ‘d’ the distance between points:

d = √(*x*2 − x1)^2^ + (*y*2 − *y*1)^2^

*x*1 and *y*1 are the coordinates of the first point and *x*2 and *y*2 of the second; x represents the horizontal position and y represents the vertical.
